# Development and validation of a multi-parametric MRI diagnostic model for differentiating hemangioma-like metastases from small (< 3 cm) hepatic hemangiomas: a size-based subgroup analysis

**DOI:** 10.1186/s12880-026-02382-4

**Published:** 2026-05-20

**Authors:** Pengrui Gao, Funing Chu, Qingcheng Meng, Chongxiao Guan, Hongkai Zhang, Yong Zhang, Jinrong Qu

**Affiliations:** 1 Department of Radiology, The Affiliated Cancer Hospital of Zhengzhou University & Henan Cancer Hospital, 127 Dongming Road, Zhengzhou, Henan 450008 China; 2 HNHC Key Laboratory of Oncology Medical Imaging Response Assessment, Henan Cancer Hospital, Zhengzhou, Henan 450008 China; 3 Department of Biological Immunotherapy, The Affiliated Cancer Hospital of Zhengzhou University & Henan Cancer Hospital, Zhengzhou, Henan 450008 China

**Keywords:** Hepatic metastasis, Hemangioma, Magnetic resonance imaging, Diagnosis, Differential, Logistic models

## Abstract

**Background:**

Hemangioma-like metastases (HM) are rare but treacherous hypervascular malignancies that mimic the imaging features of benign hepatic hemangiomas (HH), particularly when lesions are small (< 3 cm). This resemblance creates a “diagnostic grey zone,” often leading to misdiagnosis and inappropriate treatment delays. This study aims to develop and evaluate a multi-parametric MRI model to accurately distinguish small HM from HH and assess its diagnostic performance across different lesion size subgroups.

**Methods:**

This retrospective study analyzed 149 lesions (81 HMs in 37 patients and 68 HHs in 48 patients), all smaller than 3 cm. Qualitative features on T2-weighted imaging (T2WI), diffusion-weighted imaging (DWI), and dynamic contrast-enhanced (DCE) MRI were systematically evaluated. Multivariate logistic regression was employed to identify independent predictors and construct a combined diagnostic model. The calibration of the nomogram was assessed using calibration plots with 1,000 bootstrap resamples. Decision curve analysis (DCA) was performed to evaluate the clinical utility of the model by quantifying the net benefit at different threshold probabilities. The diagnostic performance was further validated in three size-based subgroups: 5–<10 mm, 10–<20 mm, and 20–<30 mm.

**Results:**

Four MRI features emerged as robust independent predictors of HM: moderately hyperintense T2WI signal (OR, 18.97; 95% CI, 3.65–98.72), heterogeneous T2WI architecture (OR, 54.28; 95% CI, 6.50–453.56), ring-like arterial enhancement (OR, 90.76; 95% CI, 10.61–776.68), and unclear delayed phase boundary (OR, 11.05; 95% CI, 1.50–81.65). The combined multi-parametric model achieved superior diagnostic performance compared to any single feature, yielding an area under the curve (AUC) of 0.981, with a sensitivity of 95.1% and a specificity of 97.1%. Subgroup analysis revealed that while the diagnostic accuracy of individual features (especially DWI) improved with increasing lesion size, the combined model maintained high diagnostic stability even in the challenging 5–<10 mm subgroup.

**Conclusion:**

The proposed multi-parametric MRI model offers an effective, non-invasive tool for differentiating small HMs from HHs. The presence of heterogeneous T2WI signal and ring-like arterial enhancement should trigger high suspicion of malignancy, even in sub-centimeter lesions. This model has the potential to aid clinicians in risk stratification and may help reduce unnecessary biopsies.

**Supplementary Information:**

The online version contains supplementary material available at 10.1186/s12880-026-02382-4.

## Introduction

Hepatic metastases are the most common malignant hepatic lesions [1], while hepatic hemangioma is the most common benign tumor of the liver, with an incidence of 3%-20% in the population according to autopsy reports [2–4]. While the majority of hepatic metastases present with typical imaging features (e.g., “washout” or rim enhancement) that are easily distinguishable from the “lightbulb” sign of hemangiomas, a subset of hypervascular metastases—termed hemangioma-like metastases (HM)—poses a significant diagnostic challenge [5, 6].

HMs are characterized by marked arterial phase enhancement and persistent hyperintensity during the portal venous and delayed phases, closely mimicking the hemodynamic patterns of classic HH [7, 8]. This diagnostic dilemma is exacerbated when lesions are small (< 3 cm). In this size range, the characteristic morphological features of metastases may be underdeveloped, and the small volume limits the spatial resolution of imaging, creating a “diagnostic grey zone” for radiologists. Previous literature emphasizes that the diagnostic accuracy for small (< 3 cm) or sub-centimeter hypervascular liver lesions drops significantly compared to larger lesions. For instance, Bae et al. demonstrated that the sensitivity of contrast-enhanced MRI for sub-centimeter HCCs was only 63%, compared to 91% for larger lesions [9]. Similarly, Kim et al. [10] found that conventional dynamic MRI had a sensitivity of just 72% for small hypervascular HCCs, with only 43% of sub-centimeter lesions exhibiting the typical washout appearance. While comparable multi-parametric MRI studies incorporating diffusion-weighted imaging (DWI) or hepatobiliary contrast agents have demonstrated improved overall diagnostic accuracies often exceeding 90%, the misdiagnosis risk remains disproportionately high for small lesions due to atypical enhancement patterns, overlapping hemodynamics, and spatial resolution limits. Misdiagnosing an HM as a benign hemangioma can lead to disastrous consequences, such as delayed oncologic intervention or inappropriate exclusion from curative surgery [11, 12]. Conversely, misdiagnosing a hemangioma as a metastasis may result in unnecessary anxiety and invasive biopsies.

Although ultrasound, CT, and FDG-PET/CT all have utility in diagnosing HM and HH, they possess certain limitations. Ultrasound’s diagnostic accuracy can be affected by operator skills and exploration depth, and it may struggle to accurately characterize smaller lesions. The diagnostic capability of CT can also be influenced by factors like the lesion’s size and location. FDG-PET/CT is similarly impacted by factors such as lesion size and glucose metabolism level, and it has a known false positive and false negative rate [13, 14]. Magnetic Resonance Imaging (MRI), particularly with hepatobiliary contrast agents (e.g., gadoxetate disodium), has improved diagnostic accuracy [15, 16]. However, the high cost, transient respiratory motion artifacts, and limited availability of these specific agents in some centers hinder their routine use for every incidental lesion. Furthermore, while diffusion-weighted imaging (DWI) has shown promise [17], considerable overlap in ADC values between HMs and HHs limits its utility as a standalone marker.

While previous studies have explored MRI-based differentiation of liver lesions, many suffered from selection bias by mixing hypervascular and hypovascular metastases, or by analyzing a wide range of tumor sizes without a specific focus on sub-centimeter lesions. Consequently, specific diagnostic criteria for small (< 3 cm) HMs remain underdefined. Furthermore, the rationale for deliberately focusing only on HM versus HH, while excluding other hypervascular benign lesions such as focal nodular hyperplasia (FNH) and hepatocellular adenoma (HCA), is rooted in clinical pragmatism. HM and HH share almost identical hemodynamic patterns on standard extracellular contrast-enhanced MRI (rapid arterial enhancement and prolonged contrast retention), representing the most frequent and challenging diagnostic dilemma. In contrast, FNH and HCA typically present with distinct morphological hallmarks (e.g., central scar, intralesional fat or hemorrhage) or specific patient demographics, and their definitive diagnosis often follows a different clinical algorithm.

The novelty of our study lies in addressing this specific diagnostic ‘grey zone’ through a size-stratified analysis (down to 5 mm). There is an urgent clinical need for a robust, highly accessible diagnostic tool. Therefore, we aimed to develop and validate a multi-parametric diagnostic nomogram integrating morphological, diffusion, and perfusion features based purely on standard extracellular contrast-enhanced MRI, providing a broadly applicable solution for the most challenging clinical scenarios.

## Methods

### Study population

**Fig. 1:**
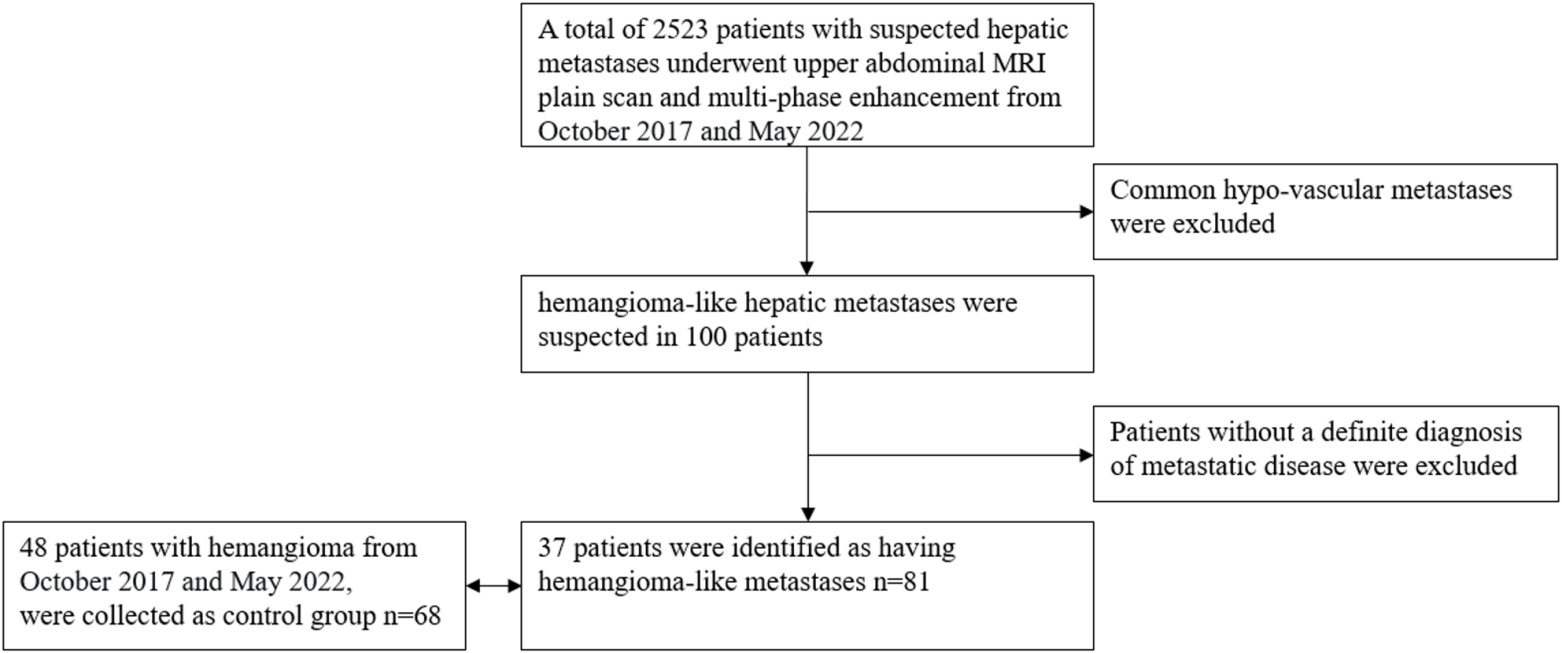
Study flowchart. The flowchart illustrates the inclusion and exclusion criteria for the study cohort. A total of 149 lesions (81 hemangioma-like metastases and 68 hepatic hemangiomas) in 85 patients were finally included for analysis

This retrospective study was approved by the Institutional Ethics Committee of Henan Cancer Hospital, and the requirement for informed consent was waived due to the retrospective nature of the analysis. A total of 149 hepatic lesions in 85 patients were identified between October 2017 and May 2022 (Fig. 1). The inclusion criteria were as follows: (1) hepatic lesions confirmed as either hemangioma-like metastasis (HM) or hepatic hemangioma (HH) by pathology (*n* = 71) or rigorous imaging follow-up criteria (stability > 12 months) (*n* = 78); (2) maximum lesion diameter < 3.0 cm; and (3) availability of complete multi-parametric MRI data, including T2-weighted imaging (T2WI), diffusion-weighted imaging (DWI), and dynamic contrast-enhanced (DCE) sequences. Exclusion criteria included: (1) lesions < 5 mm in diameter; (2) severe motion artifacts affecting image interpretation; and (3) history of prior local treatment (e.g., radiofrequency ablation) for the target lesion. The final cohort consisted of 81 HMs (in 37 patients) and 68 HHs (in 48 patients). The primary malignancies for the HM group are detailed in Supplementary Table 1.

### MRI protocol

All MRI examinations were performed using a 3.0-T scanner (MAGNETOM Skyra; Siemens Healthineers, Erlangen, Germany) equipped with an 18-channel body matrix coil. The standardized protocol included respiratory-triggered T2WI, DWI (b-values: 50 and 800 s/mm²), and DCE-MRI using a T1-weighted volumetric interpolated breath-hold examination (TWIST-VIBE) sequence. The contrast agent (Gd-DTPA, 0.1 mmol/kg) was administered intravenously at a rate of 2.5 mL/s. Detailed acquisition parameters are summarized in Supplementary Table 2.

### Image analysis

Three radiologists with over 5 years of experience in abdominal imaging, blinded to clinical information and final diagnoses, independently reviewed the images. Inter-observer agreement was calculated based on these initial independent evaluations. Disagreements were resolved through consensus discussion. Qualitative features evaluated included: (1) T2WI signal intensity, categorized as “markedly hyperintense” (isointense to cerebrospinal fluid) or “moderately hyperintense” (2) T2WI architecture (homogeneous vs. heterogeneous); (3) DWI signal pattern, categorized as uniform high signal or peripheral high signal (“target sign”); (4) Arterial phase enhancement, classified as ring-like, peripheral nodular, or diffuse enhancement; and (5) Delayed phase characteristics, specifically assessing the presence of contrast filling and boundary clarity (clear vs. unclear).

### Statistical analysis

Statistical analyses were performed using SPSS software (version 24.0; IBM Corp., Armonk, NY) and R software (version 4.2.1; The R Foundation for Statistical Computing). Given the strict inclusion criteria requiring complete multiparametric MRI data, the final analyzed cohort had 100% data completeness. Therefore, a complete-case analysis approach was inherently applied, and no missing data imputation methods (e.g., multiple imputation) were required. Continuous variables were expressed as mean ± standard deviation (SD) and compared using the independent t-test. Categorical variables were compared using the Chi-square test or Fisher’s exact test. Inter-observer agreement was assessed using the intraclass correlation coefficient (ICC).

Univariate logistic regression analysis was initially conducted to identify potential predictors of HM, and variables with *P* < .05 were selected for further analysis. To assess potential redundancy among the MRI features, multicollinearity was evaluated using the Variance Inflation Factor (VIF), with a VIF < 5 considered indicative of no severe collinearity. Furthermore, to address the issue of quasi-complete separation (sparse data bias) observed in standard maximum likelihood estimation, Firth’s penalized likelihood logistic regression was employed for the multivariable analysis. This shrinkage technique effectively reduces overfitting and provides mathematically stable odds ratios (OR) and 95% confidence intervals (CI). As this was a retrospective study, a formal a priori power analysis was not conducted. Instead, the sample size was determined by the total number of eligible cases during the study period. To ensure the reliability of the multivariate logistic regression model and minimize the risk of overfitting, we adhered to the “events per variable” (EPV) guideline. With 81 events (HM lesions) and 4 independent predictors included in the final multivariable model, our EPV was approximately 20, which robustly exceeds the widely recommended minimum threshold of 10. Based on the multivariate analysis results, a diagnostic nomogram was constructed using the rms package in R. The discriminative performance of the model was quantified using the area under the receiver operating characteristic (ROC) curve (AUC). The calibration of the nomogram was assessed using calibration plots with 1,000 bootstrap resamples. Decision curve analysis (DCA) was performed to evaluate the clinical utility of the model by quantifying the net benefit at different threshold probabilities. Finally, a subgroup analysis stratified by lesion size (5–<10 mm, 10–<20 mm, and 20–<30 mm) was performed to evaluate the stability of the diagnostic features and the combined model. A two-sided P value < 0.05 was considered statistically significant.

## Results

### Patient and lesion characteristics

There were no significant differences in age (*P* = .848) or maximum lesion diameter (*P* = .694) between the HM and HH groups (HM: 13.8 ± 6.7 mm vs. HH: 14.2 ± 6.0 mm). A significant female predominance was observed in the HM group (*P* = .006), consistent with the high prevalence of breast cancer (23/37) as the primary tumor site in this cohort. The inter-reader agreement for all MRI features was excellent, with ICC values ranging from 0.859 to 0.956 (Supplementary Table 3).

### Univariate and multivariate analysis of MRI features

Univariate analysis revealed significant differences between HM and HH across all evaluated imaging parameters (Table 1). Representative cases of HM and HH are shown in Supplementary Figs. 1–3. Specifically, HMs were characterized by moderately hyperintense T2WI signal (82.7%), heterogeneous T2WI architecture (80.2%), ring-like arterial enhancement (75.3%), and unclear boundaries in the delayed phase (60.5%) (all *P* < 0.001). Collinearity diagnostics confirmed no significant multicollinearity among the selected predictors, with all VIF values well below 5 (ranging from 1.35 to 1.63). Subsequent multivariable analysis using Firth’s penalized logistic regression identified four independent predictors of HM: moderately hyperintense T2WI signal (OR, 18.97; 95% CI, 3.65–98.72; *P* = 0.0005), heterogeneous T2WI architecture (OR, 54.28; 95% CI, 6.50–453.56; *P* = 0.0002), ring-like arterial enhancement (OR, 90.76; 95% CI, 10.61–776.68; *P* < 0.001), and unclear delayed phase boundary (OR, 11.05; 95% CI, 1.50–81.65; *P* = 0.018) (Table 2).

**Table 1:** Comparison of clinical characteristics and MRI signal characteristics between hemangioma-like metastasis (HM) and hepatic hemangioma (HH)

Characteristics	HM (*n* = 81, *P* = 37)	HH (*n* = 68, *P* = 48)	χ2 /t Value	*P* value
**Gender**			7.682	0.006
Male	9	26		
Female	28	22		
**Age**	53.0 ± 10.5	52.6 ± 8.7	0.193	0.848
**Number of lesions**			7.259	0.007
Solitary	10	27		
Multiple	27	21		
**Maximum diameter (mm)**	13.8 ± 6.7	14.2 ± 6.0	-0.413	0.694
**T2WI signal intensity**			77.558	< 0.001
Slightly high signal	67	7		
Obvious high signal	14	61		
**T2WI signal characteristic**			72.438	< 0.001
Homogenous	16	61		
Heterogeneous	65	7		
**DWI signal characteristic**			33.899	< 0.001
Uniform high signal	39	63		
Edge higher signal	42	5		
**Enhanced type in arterial phase**				
Ring-like enhancement	61	4	77.450	< 0.001
Peripheral nodular or total enhancement	20	64		
**Contrast filling in delayed phase**			12.119	< 0.001
Yes	53	61		
No	28	7		
**Boundary in delayed phase**			42.381	< 0.001
Clear	32	62		
Unclear	49	6		
**Shape**			1.845	0.174
Round	70	53		
Irregular	11	15		

**Table 2:** Univariable and multivariable logistic regression of factors in differentiating hemangioma-like metastasis from hemangioma

MR imaging features	Univariable OR (95%CI)	*P* Value	Multivariable OR (95%CI)	*P* Value
T2WI signal intensity	41.7 (15.8-110.2)	< 0.001	18.97 (3.65–98.72)	0.001
T2WI signal characteristic	35.5 (13.6–91.9)	< 0.001	54.28 (6.50-453.56)	< 0.001
DWI signal characteristic	13.6 (4.9–37.2)	< 0.001		
Enhanced type in arterial phase	48.8 (15.8–151.0)	< 0.001	90.76 (10.61-776.68)	< 0.001
Contrast filling in delayed phase	4.6 (1.9–11.4)	0.001		
Boundary in delayed phase	15.8 (6.1–40.9)	< 0.001	11.05 (1.50-81.65)	0.019
Shape	1.8 (0.8–4.2)	0.178		

**Abbreviations**: OR= Odds Ratio. Note: Multivariable ORs and 95% CIs were calculated using Firth’s penalized likelihood logistic regression to account for sparse data bias

### Development and validation of the diagnostic nomogram

**Fig. 2:**
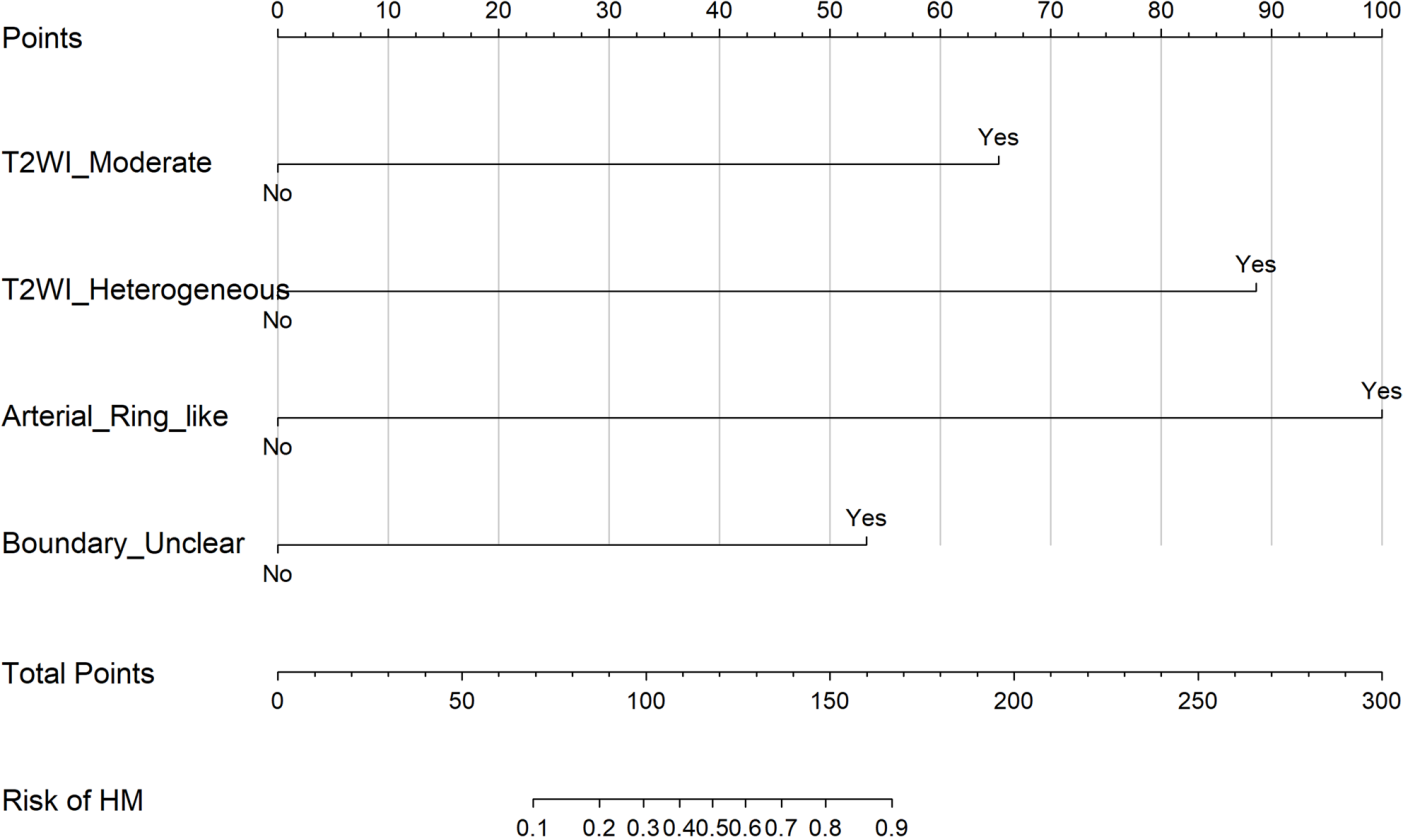
Diagnostic Nomogram for predicting the probability of Hemangioma-like Metastasis (HM). The nomogram incorporates four independent MRI predictors derived from Firth’s penalized likelihood logistic regression: T2WI moderate hyperintensity, T2WI heterogeneous architecture, arterial ring-like enhancement, and unclear delayed boundary

**Fig. 3:**
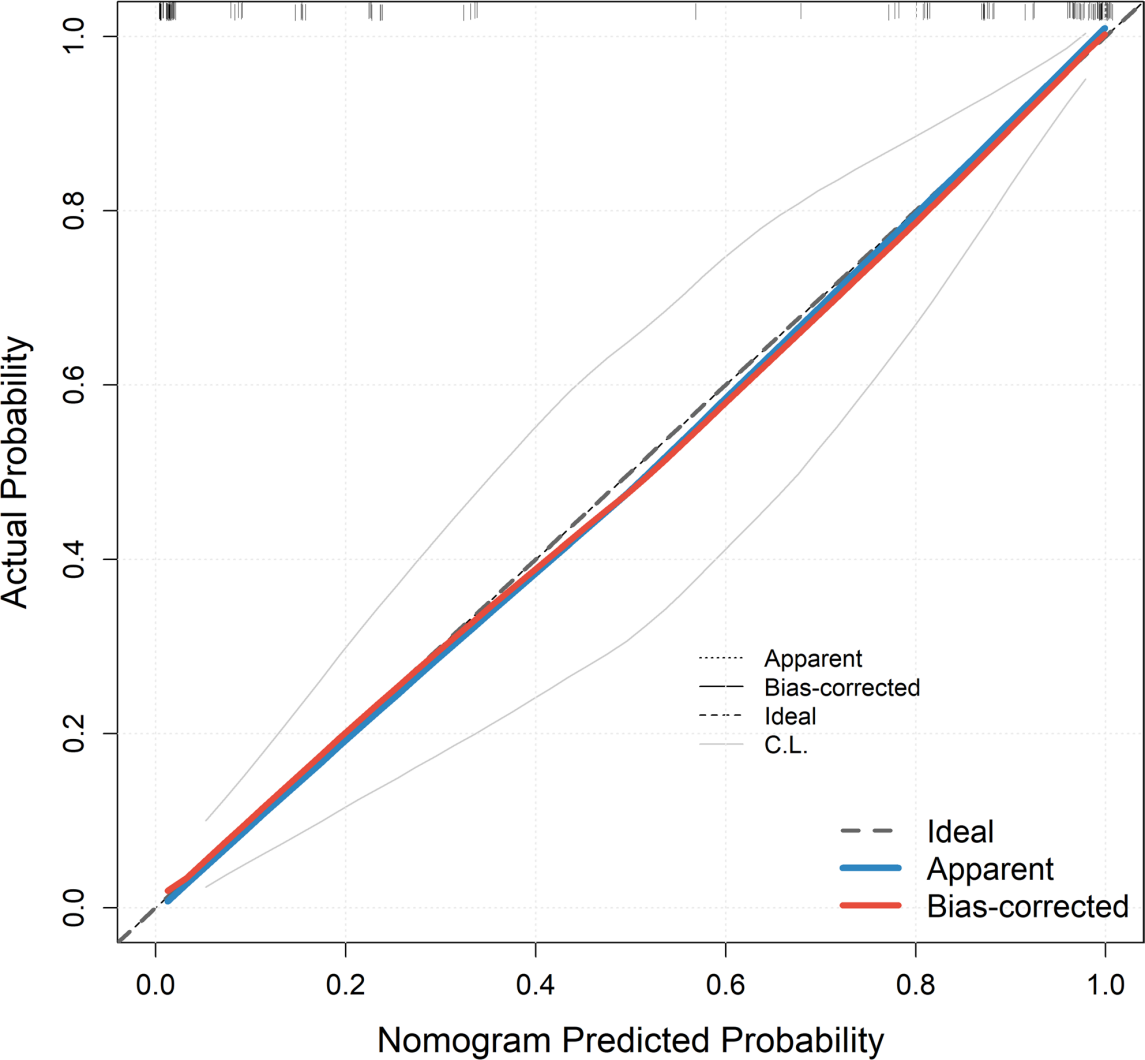
Calibration curve of the diagnostic nomogram for predicting hepatic metastasis (HM). The x-axis represents the nomogram-predicted probability, and the y-axis represents the actual observed probability. The gray dashed line indicates perfect calibration of an ideal model. The blue solid line represents the apparent performance of the nomogram, and the red solid line represents the bias-corrected performance (using 1,000 bootstrap resamples). The nomogram demonstrates excellent calibration as both lines closely align with the ideal reference line

**Fig. 4:**
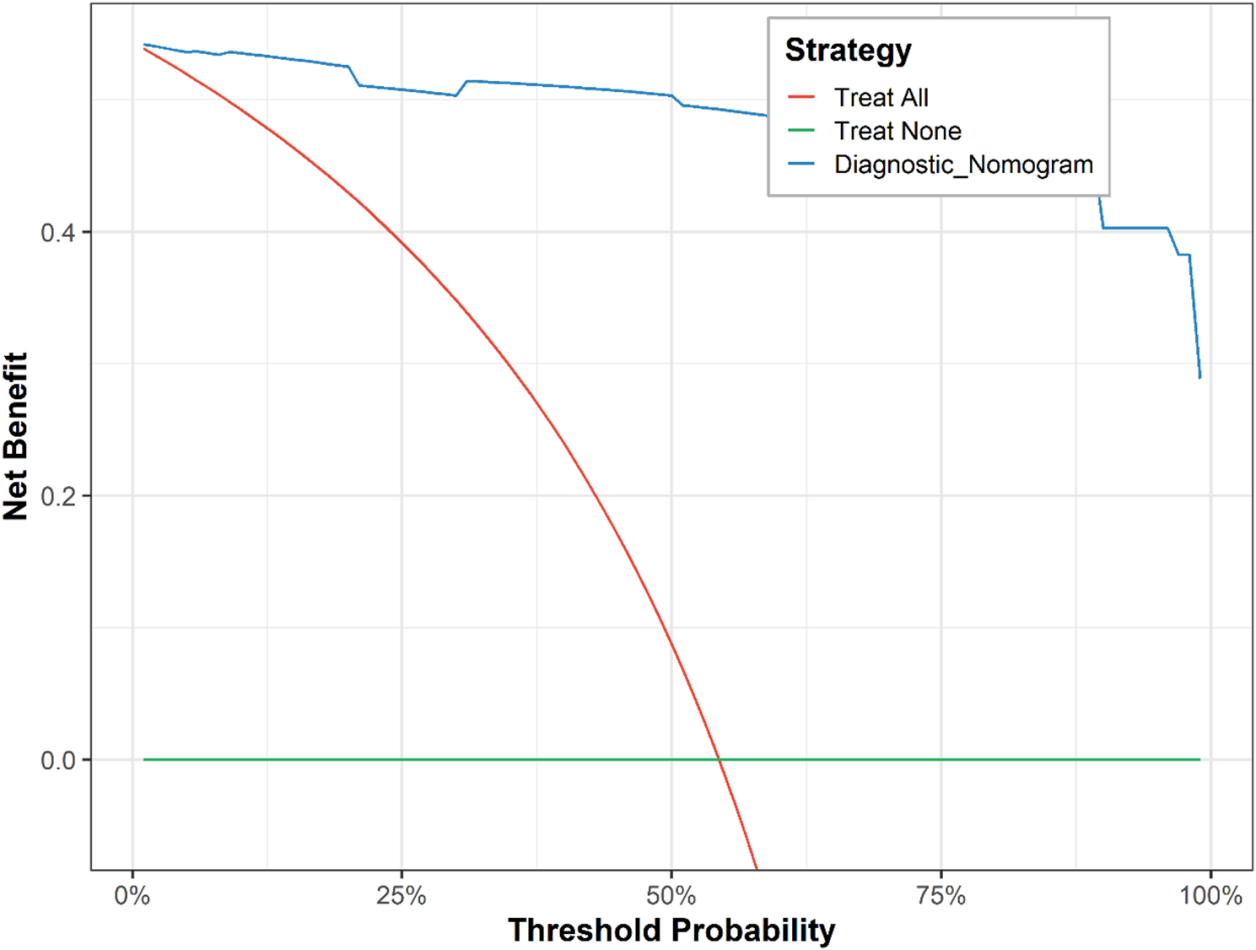
Decision curve analysis (DCA) of the diagnostic nomogram. The y-axis represents the net clinical benefit. The blue solid line represents the diagnostic nomogram. The red solid line represents the “Treat All” strategy, and the green solid line represents the “Treat None” strategy. The nomogram provides a higher net benefit than either default strategy across a wide range of threshold probabilities, indicating its substantial clinical utility

A diagnostic nomogram incorporating the four independent predictors was established to predict the probability of HM (Fig. 2). The combined model demonstrated excellent discriminative ability, yielding an AUC of 0.981 (95% CI, 0.960–0.999), significantly higher than that of any single feature (AUC range: 0.758–0.862) (*P* < 0.001) (Table 3). The calibration curve (Fig. 3) demonstrated good agreement between the nomogram-predicted probabilities and the actual observations. Decision curve analysis (Fig. 4) indicated that using the nomogram to predict HM provided a greater net benefit than “treat-all” or “treat-none” strategies across a wide range of threshold probabilities, confirming its clinical utility.

**Table 3:** AUCs of single feature and combined all the features in differentiating hemangioma-like metastasis from hemangioma

MR imaging features	AUC	Sensitivity	Specificity	*P* value
T2WI signal intensity	0.862	82.7%	89.7%	< 0.001
T2WI signal characteristic	0.850	80.2%	89.7%	< 0.001
DWI signal characteristic	0.722	51.9%	92.6%	< 0.001
Enhanced type in arterial phase	0.847	75.3%	94.1%	< 0.001
Boundary in delayed phase	0.758	60.5%	91.2%	< 0.001
Combined features	0.981	95.1%	97.1%	< 0.001

### Subgroup analysis by lesion size

The diagnostic performance of individual MRI features varied with lesion size (Tables 4 and 5). The subgroup sample sizes were as follows: 5–<10 mm (HM: *n* = 30, HH: *n* = 18), 10–<20 mm (HM: *n* = 35, HH: *n* = 34), and 20–<30 mm (HM: *n* = 16, HH: *n* = 16). In the smallest subgroup (5–<10 mm), DWI signal characteristics showed no statistically significant difference between HM and HH (*P* = 0.110), with a sensitivity of only 33.3%. In contrast, T2WI signal intensity and arterial enhancement patterns remained robust predictors in this subgroup (AUC 0.833 and 0.783, respectively). As lesion size increased to 10–<20 mm and 20–<30 mm, the diagnostic performance of all parameters improved. Notably, the sensitivity of the DWI “target sign” increased from 33.3% in the < 10 mm group to 81.3% in the 20–30 mm group (*P* < 0.001). Despite the variation in individual features, the combined multi-parametric model maintained high and stable diagnostic accuracy across all subgroups. Statistical comparisons using DeLong’s test revealed no significant differences in the AUCs of the combined model between the 5–<10 mm, 10–<20 mm, and 20–<30 mm subgroups (all *P* > .05), further demonstrating the robustness of the nomogram across different lesion sizes.

**Table 4:** Comparison of MR imaging features between hemangioma-like metastasis and hepatic hemangioma

MR imaging features	≥ 5 and<10 mm				≥ 10 and < 20 mm				≥ 20 and < 30 mm			
	HHM (*n* = 30)	HH (*n* = 18)	χ2 Value	*P* value	HHM (*n* = 35)	HH (*n* = 34)	χ2 Value	*P* value	HHM (*n* = 16)	HH (*n* = 16)	χ2 Value	*P* value
**T2WI signal intensity**			20.571	< 0.001			34.932	< 0.001				< 0.001
Slight high signal	25	3			29	4			13	0		
Obvious high signal	5	15			6	30			3	16		
**T2WI signal characteristic**			11.063	0.001			36.486	< 0.001			21.208	< 0.001
Homogenous	12	16			3	30			2	15		
Heterogeneous	18	2			32	4			14	1		
**DWI signal characteristic**			4.914	0.027			20.749	< 0.001				< 0.001
Uniform high signal	20	17			15	30			3	16		
Edge higher signal	10	1			20	4			13	0		
**Enhanced type in arterial phase**			14.475	< 0.001			34.449	< 0.001				< 0.001
Ring-like enhancement	22	3			25	1			12	0		
Peripheral nodular or total enhancement	8	15			10	33			4	16		
**Boundary in delayed phase**			10.000	0.002			23.081	< 0.001			11.221	0.001
Clear	15	17			11	30			6	15		
Unclear	15	1			24	4			10	1		

**Table 5:** Sensitivity and specificity of individual MR imaging features for differentiating hemangioma-like metastasis from hemangioma

MR imaging features	≥ 5 and<10 mm				≥ 10 and < 20 mm				≥ 20 and <30 mm			
	AUC	Sensitivity	Specificity	*P* value	AUC	Sensitivity	Specificity	*P* value	AUC	Sensitivity	Specificity	*P* value
T2WI signal intensity	0.833 (0.724–0.942)	83.3 (66.4–92.7)	83.3 (60.8–94.2)	< 0.001	0.855 (0.773–0.938)	82.9 (67.3–91.9)	88.2 (73.4–95.3)	< 0.001	0.906 (0.811–1.000)	81.3 (57.0–93.4)	100.0 (80.6–100.0)	< 0.001
T2WI signal characteristic	0.744 (0.631–0.858)	60.0 (42.3–75.4)	88.9 (67.2–96.9)	0.005	0.898 (0.827–0.970)	91.4 (77.6–97.0)	88.2 (73.4–95.3)	< 0.001	0.906 (0.806–1.000)	87.5 (64.0–96.5)	93.8 (71.7–98.9)	< 0.001
DWI signal characteristic	0.639 (0.539–0.738)	33.3 (19.2–51.2)	94.4 (74.2–99.0)	0.110	0.727 (0.629–0.825)	57.1 (40.9–72.0)	88.2 (73.4–95.3)	0.001	0.906 (0.811–1.000)	81.3 (57.0–93.4)	100.0 (80.6–100.0)	< 0.001
Enhanced type in arterial phase	0.783 (0.666–0.900)	73.3 (55.6–85.8)	83.3 (60.8–94.2)	0.125	0.842 (0.762–0.922)	71.4 (54.9–83.7)	97.1 (85.1–99.5)	0.031	0.875 (0.769–0.981)	75.0 (50.5–89.8)	100.0 (80.6–100.0)	< 0.001
Boundary in delayed phase	0.722 (0.618–0.826)	50.0 (33.2–66.8)	94.4 (74.2–99.0)	< 0.001	0.784 (0.690–0.878)	68.6 (52.0–81.4)	88.2 (73.4–95.3)	< 0.001	0.781 (0.649–0.914)	62.5 (38.6–81.5)	93.8 (71.7–98.9)	0.007

## Discussion

In this study, we developed and validated a multi-parametric MRI nomogram for distinguishing hemangioma-like metastases (HM) from hepatic hemangiomas (HH) in small lesions (< 3 cm). While typical large metastases are easily identifiable, sub-centimeter hypervascular lesions remain a diagnostic “grey zone,” often necessitating invasive biopsies or causing significant anxiety [18]. Our model, integrating T2WI signal intensity and architecture, arterial enhancement pattern, and delayed phase boundary, achieved an outstanding AUC of 0.981. Crucially, our subgroup analysis revealed that while single parameters (like DWI) lose sensitivity in lesions < 10 mm, the combined model maintains robust diagnostic accuracy. This highlights the necessity of a multi-parametric approach in the era of early cancer detection [19].

The arterial phase enhancement pattern emerged as the most powerful independent predictor (OR = 90.76; 95% CI, 10.61–776.68). We found that “ring-like enhancement” is a highly specific marker for HM, contrasting with the “peripheral nodular enhancement” typical of HH. Pathologically, this ring-like appearance in metastases corresponds to a rim of viable, hypervascular tumor cells at the periphery, surrounding a center of relative ischemia, necrosis, or fibrosis. Even in small lesions, this “central-peripheral” biological discrepancy exists [20]. In contrast, hemangiomas are composed of blood-filled vascular lakes. In small hemangiomas (< 3 cm), the enhancement is often “flash-filling” or “nodular” but rarely presents as a complete, thin rim with a non-enhancing center. Our findings reinforce that any “ring” pattern in a small liver nodule should be considered malignant until proven otherwise, strongly suggesting malignancy, although a certain degree of diagnostic overlap with atypical hemangiomas should be acknowledged.

T2-weighted imaging remains a cornerstone for liver lesion characterization. Consistent with classic teaching, 89.7% of HHs in our cohort exhibited the “lightbulb sign” (markedly hyperintense and homogeneous), reflecting their fluid-rich cystic spaces with slow flow. However, the real value lies in the subtle features of HM. We observed that 80.2% of HMs were heterogeneous and only moderately hyperintense. This heterogeneity likely reflects the complex internal architecture of metastases, which contain a mix of viable tumor cells, desmoplastic stroma, and microscopic hemorrhage. Therefore, radiologists should be wary of the term “hemangioma-like.” While HMs are hypervascular like hemangiomas [5, 21], their T2WI signal is rarely as “pure” or “bright.” A “dirty,” moderately bright T2 signal should trigger suspicion.

A unique contribution of our study is the size-stratified analysis of DWI. While the “target sign” (peripheral high signal) on DWI is a well-known feature of liver metastases, our data showed a stark drop in sensitivity for lesions < 10 mm (33.3%) compared to those 20–30 mm (81.3%). This phenomenon can be attributed to the “partial volume effect” and limited spatial resolution of DWI sequences. In sub-centimeter lesions, the subtle central vs. peripheral signal difference is blurred, merging into a uniform high signal that mimics a hemangioma. This finding is clinically critical: it cautions radiologists not to rely solely on DWI for very small lesions. If a < 10 mm lesion lacks the “target sign” on DWI, it does not exclude malignancy. In such cases, the arterial enhancement pattern (included in our nomogram) carries more diagnostic weight.

We identified “unclear boundary in the delayed phase” as a significant predictor for HM (OR = 11.05; 95%CI, 1.50-81.65). Hepatic hemangiomas are typically well-circumscribed due to a fibrous pseudocapsule or sharp interface with hepatocytes. In contrast, metastases, particularly aggressive ones, often exhibit infiltrative growth into the surrounding liver parenchyma, leading to an indistinct margin known as the “halo sign” or simply an ill-defined border on delayed images. This subtle sign is often overlooked but serves as a valuable clue when arterial and T2WI features are equivocal.

Moving beyond statistical significance, we focused on clinical utility. To our knowledge, this is one of the first studies to apply Decision Curve Analysis (DCA) to this specific differential diagnosis. The DCA demonstrated that using our nomogram provides a higher net benefit than “treat-all” or “treat-none” strategies across a wide range of threshold probabilities. Practically, this means that by using our scoring system, clinicians can confidently identify high-risk patients for biopsy while safely observing those with low scores, thereby reducing the rate of unnecessary invasive procedures—a crucial consideration given the risks of liver biopsy.

We acknowledge a predominance of breast cancer patients (23/37) in our HM cohort. While this reflects a selection bias, it also represents a high-impact clinical scenario. Breast cancer is known to produce hypervascular liver metastases that frequently mimic hemangiomas [22]. Therefore, our study should be interpreted as particularly applicable to the surveillance of breast cancer survivors. For this high-risk population, distinguishing a benign “incidentaloma” from a solitary metastasis determines whether the patient remains disease-free or undergoes metastasectomy. Our model is tailored to answer this precise, high-stakes question.

Our study has several limitations that warrant acknowledgment. First, it is retrospective and single-center in design, which may introduce selection bias. Second, although 71 lesions were pathologically confirmed, the remaining 78 benign hemangiomas were diagnosed by imaging stability (> 12 months), a standard clinical practice that nevertheless carries a minor risk of misclassification bias if rare indolent malignancies mimic stability. Third, 149 lesions were obtained from 85 patients, resulting in within-patient correlation. While Generalized Estimating Equations are typically preferred, the presence of quasi-complete separation necessitated Firth’s penalized logistic regression to stabilize estimates. As integrating clustering correction with Firth’s penalty is not readily supported by standard software, we treated lesions as independent observations; this may slightly underestimate standard errors and is noted as a statistical limitation. Fourth, the model lacks independent external validation, which limits generalizability. Fifth, we relied on qualitative MRI descriptors (despite excellent inter-reader agreement, ICC 0.859–0.956), which inherently carry some subjectivity. Sixth, clinical variables such as primary tumor type and oncologic treatment history were not incorporated. Finally, our cohort showed a predominance of breast cancer patients, making the nomogram particularly suited for this high-risk population but requiring further validation in cohorts dominated by other malignancies.

In terms of clinical workflow, the nomogram serves as a practical, non-invasive triaging tool: high-risk scores prompt biopsy or closer follow-up, while low-risk scores support safe observation, reducing unnecessary invasive procedures. Future multicenter studies with external validation, as well as hybrid models combining imaging features with clinical risk factors and quantitative radiomics/deep learning, are warranted to further enhance objectivity and generalizability.

In conclusion, differentiating HM from HH in lesions smaller than 3 cm is feasible and highly accurate using our multi-parametric MRI nomogram. The presence of ring-like arterial enhancement, heterogeneous T2WI signal, and an unclear delayed boundary are potent indicators of malignancy. Special caution is advised when interpreting DWI for lesions < 10 mm, where the combined model offers superior reliability.

## Electronic Supplementary Material

Below is the link to the electronic supplementary material.

Supplementary Material 1

## Data Availability

The datasets generated and analyzed during this study are not publicly available due to patient privacy restrictions but may be made available by the corresponding author upon reasonable request.

## Abbreviations

AUC: Area under the curve
DWI: Diffusion-weighted imaging
ROC: Receiver operating characteristic
T1WI: T1-weighted imaging
T2WI: T2-weighted imaging
HM: Hemangioma-like metastasis
HH: Hepatic hemangioma
ICC: Intraclass correlation coefficient
OR: Odds ratio
DCE-MRI: Dynamic contrast-enhanced magnetic resonance imaging
