# Supplementary material for: Development and validation of a multi-parametric MRI diagnostic model for differentiating hemangioma-like metastases from small (< 3 cm) hepatic hemangiomas: a size-based subgroup analysis
Source: BMC Med Imaging. 2026 May 20;26:349. doi: 10.1186/s12880-026-02382-4 (PMC13366846; doi:10.1186/s12880-026-02382-4)
Supplement: Supplementary file 1 — Supplementary Material 1 [file 12880_2026_2382_MOESM1_ESM.docx]

**Table E1. Primary tumor types and cases of hemangioma-like metastasis**

| Primary tumor types | Number of patients |
| --- | --- |
| Breast cancer | 23 |
| Pancreatic neuroendocrine tumor | 3 |
| Gastrointestinal stromal tumor | 2 |
| Lung adenocarcinoma | 2 |
| Colorectal adenocarcinoma | 2 |
| Small cell lung carcinoma | 1 |
| Esophageal adenocarcinoma | 1 |
| Gastric adenocarcinoma | 1 |
| Gallbladder adenocarcinoma | 1 |
| Bile duct adenocarcinoma | 1 |

**Table E2. MRI protocol**

| Sequences | TR/TE (ms) | Slice thickness (mm) | Flip angle (°) | FOV | b value  (s/mm^2^) | NEX |
| --- | --- | --- | --- | --- | --- | --- |
| T1WI | 4.34/1.34 | 2.5 | 9 | 250 × 320 | — | 1 |
| T2WI(TSE) | 4000~8000/85 | 5.5 | 140 | 240 × 320 | — | 1 |
| DWI | 3100/55 | 5.5 | — | 112 × 136 | 50, 800 | — |
| TWIST-VIBE | 3.99/1.25 | 2.5 | 15 | 210 × 320 | — | — |

**Table E3 Consistency test of MR imaging features reviewed by three radiologists**

| MR imaging features | Radiologist 1 | Radiologist 2 | Radiologist  3 | ICC | *P* value |
| --- | --- | --- | --- | --- | --- |
| **T2WI signal intensity** |  |  |  | 0.903 | <.001 |
| Slight high signal | 74 | 68 | 72 |  |  |
| Obvious high signal | 75 | 81 | 77 |  |  |
| **T2WI signal characteristic** |  |  |  | 0.956 | <.001 |
| Homogenous | 77 | 75 | 78 |  |  |
| Heterogeneous | 72 | 74 | 71 |  |  |
| **DWI signal characteristic** |  |  |  | 0.948 | <.001 |
| Uniform high signal | 102 | 103 | 102 |  |  |
| - Edge higher signal | 47 | 46 | 47 |  |  |
| **Enhanced type in arterial phase** |  |  |  | 0.955 | <.001 |
| Ring-like enhancement | 65 | 66 | 63 |  |  |
| Peripheral nodular or total enhancement | 84 | 83 | 86 |  |  |
| **Contrast filling in delayed phase** |  |  |  | 0.904 | <.001 |
| Yes | 114 | 110 | 114 |  |  |
| No | 35 | 39 | 35 |  |  |
| **Boundary in delayed phase** |  |  |  | 0.859 | <.001 |
| Clear | 91 | 90 | 96 |  |  |
| Unclear | 58 | 59 | 53 |  |  |
| **Shape** |  |  |  | 0.895 | <.001 |
| Round | 26 | 26 | 29 |  |  |
| Irregular | 123 | 123 | 120 |  |  |

**Abbreviations:** ICC=intraclass correlation coefficient

**
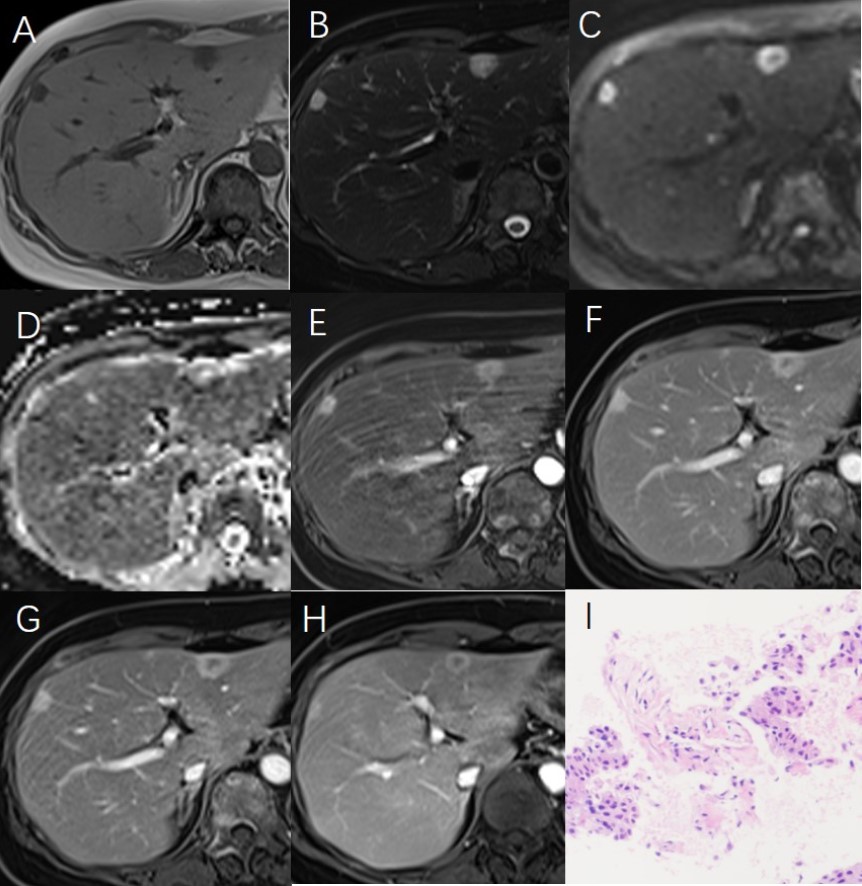
**

**Supplementary Fig. 1 Multiple hemangioma-like metastases were seen in the subcapsular region of segment II (maximum diameter 14mm) and Ⅷ (maximum diameter 11mm).** The hepatic metastases showed low signal on T1WI (A), heterogeneous high signal on T2WI (B), edge high signal on DWI (b=800s/mm2) (C), slightly ring low apparent diffusion coefficient (ADC) value on ADC map (D), significant ring-like enhancement in arterial phase (E), slightly filling in portal-venous phase (F) and delayed phase (G), the enhancement degree was no significant reduction in the delayed phase, and the boundary of the tumor was slightly rough and unclear. The lesions showed reduced size after 6 months of chemotherapy in contrast-enhanced T1WI (H). Histopathology by biopsy shows that hepatic metastasis of breast cancer; original magnification x 100 H&E Staining (I).

**
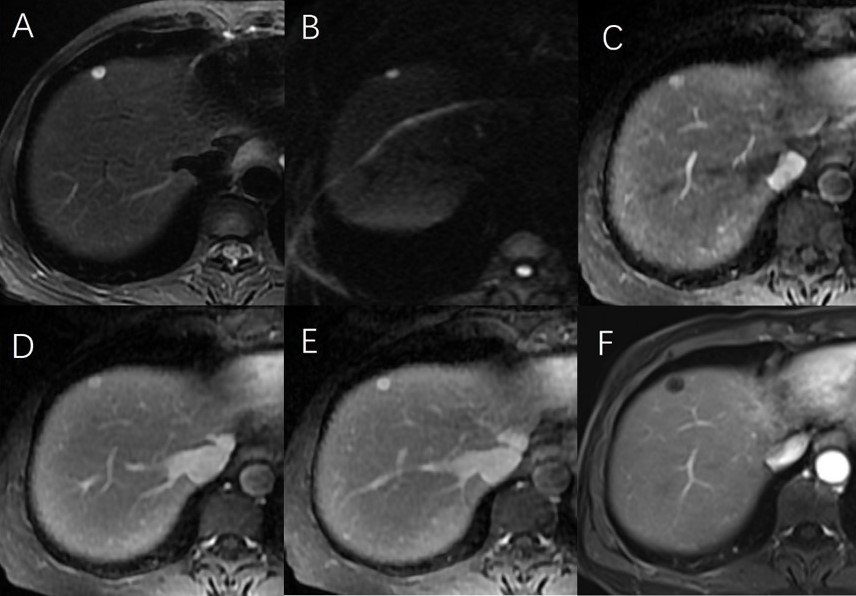
**

**Supplementary Fig. 2 Hemangioma-like metastases were seen in the subcapsular region of segment Ⅳ(maximum diameter 7mm).** The hepatic metastases showed high signal nodules on T2WI(A); Note uniform high signal high-intensity signal on diffusion-weighted image (b=800s/mm2) (B); On enhanced sequence, the nodule showed ring-like hypervascular , and no washout in portal-venous phase and delayed phase (C, D, E). Contrast-enhanced T1WI after 6 months showed the size enlarged and the intensity reduced after chemotherapy for duodenal stromal tumor(F).

**
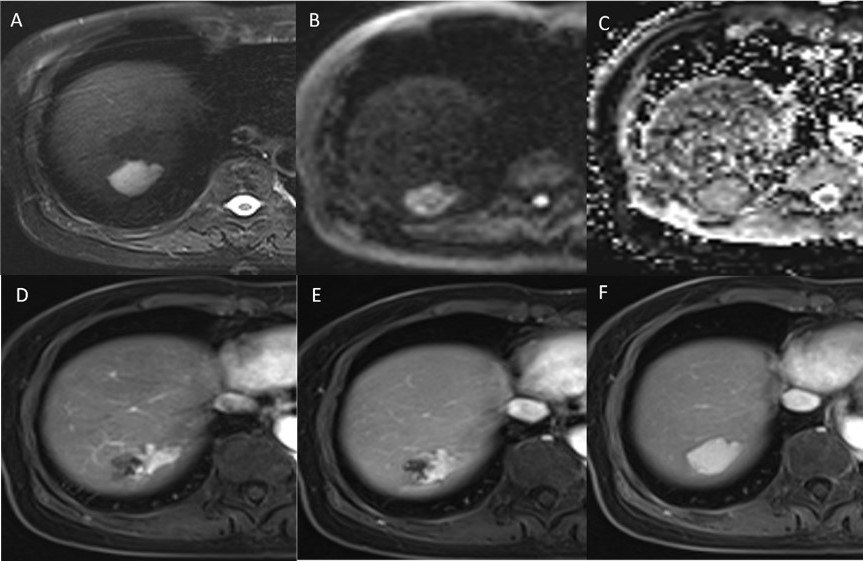
**

**Supplementary Fig. 3 Hepatic hemangiomas was seen in the subcapsular region of segment Ⅶ (maximum diameter 29mm).**  The lesions showed obvious high signal intensity on DWI (b=50，800s/mm2) and T2WI(A, B); The hemangiomas showed sightly high apparent diffusion coefficient (ADC) value on the ADC map(C). On enhanced sequence, peripheral nodular enhancement in the arterial phase with centripetal filling in the portal venous phase and complete filling in the delayed phase. The boundary of the tumor was clear (D, E, F).
